# Neuropsychiatric Lyme Disease and Vagus Nerve Stimulation

**DOI:** 10.3390/antibiotics12091347

**Published:** 2023-08-22

**Authors:** Nicholas Biniaz-Harris, Mara Kuvaldina, Brian A. Fallon

**Affiliations:** 1 Lyme & Tick-Borne Diseases Research Center at Columbia University Irving Medical Center, 1051 Riverside Drive, New York, NY 10032, USA; nb2955@cumc.columbia.edu (N.B.-H.); mk4480@cumc.columbia.edu (M.K.); 2 Department of Psychiatry, Columbia University Irving Medical Center, New York, NY 10032, USA; 3 New York State Psychiatric Institute, 1051 Riverside Drive, New York, NY 10032, USA

**Keywords:** Lyme disease, borreliosis, tick-borne illness, neuroinflammation, post-treatment Lyme disease syndrome, vagus nerve stimulation

## Abstract

Lyme disease, the most common tick-borne disease in the United States, is caused by infection with the spirochete *Borrelia burgdorferi*. While most patients with acute Lyme disease recover completely if treated with antibiotics shortly after the onset of infection, approximately 10–30% experience post-treatment symptoms and 5–10% have residual symptoms with functional impairment (post-treatment Lyme disease syndrome or PTLDS). These patients typically experience pain, cognitive problems, and/or fatigue. This narrative review provides a broad overview of Lyme disease, focusing on neuropsychiatric manifestations and persistent symptoms. While the etiology of persistent symptoms remains incompletely understood, potential explanations include persistent infection, altered neural activation, and immune dysregulation. Widely recognized is that new treatment options are needed for people who have symptoms that persist despite prior antibiotic therapy. After a brief discussion of treatment approaches, the article focuses on vagus nerve stimulation (VNS), a neuromodulation approach that is FDA-approved for depression, epilepsy, and headache syndromes and has been reported to be helpful for other diseases characterized by inflammation and neural dysregulation. Transcutaneous VNS stimulates the external branch of the vagus nerve, is minimally invasive, and is well-tolerated in other conditions with few side effects. If well-controlled double-blinded studies demonstrate that transcutaneous auricular VNS helps patients with chronic syndromes such as persistent symptoms after Lyme disease, taVNS will be a welcome addition to the treatment options for these patients.

## 1. Introduction

Lyme disease, the most common tick-borne disease in the United States, is caused by infection with the spirochete *Borrelia burgdorferi*. In 2019 alone, 34,945 confirmed or probable cases of Lyme disease were reported to the CDC [1]. These figures are thought to be drastic underestimates of the actual incidence because most cases are not reported to the CDC. An analysis of insurance records in the USA from 2010–2018 found that approximately 476,000 Americans are diagnosed and treated for Lyme disease every year [2,3].

Signs and symptoms of Lyme disease stem from the host’s inflammatory response to the microbe. The most characteristic early sign of Lyme disease is the erythema migrans rash, which occurs in 60–70% of patients. It is typically expanding, painless, and greater than two inches in diameter. It most often does not have a “bull’s eye” appearance. The early disseminated and late disseminated stages of Lyme disease impact multiple organ systems, including the skin (multiple erythema migrans rashes), peripheral nervous system, central nervous system (e.g., meningitis, radiculitis, encephalitis), joints (arthritis), and heart (e.g., heart block, carditis). However, up to 20% of patients with Lyme disease may lack these objective signs and therefore not be recognized by clinicians as having Lyme disease [4]. These patients instead may present with a constellation of viral-like symptoms including myalgia and fatigue.

The two most frequently used diagnostic tests for Lyme disease are the serologic assays—ELISA (whole cell sonicate, C6, C10/VIsE) and western blot. When used together, these tests have a sensitivity of 30–50% at the time of the EM rash and 70–90% at the time of acute neurologic Lyme disease. Testing of spinal fluid for acute central neurologic Lyme disease typically reveals mildly elevated protein and pleocytosis as well as a higher concentration of *Borrelia burgdorferi* antibodies in the cerebrospinal fluid (CSF) compared to the serum (“intrathecal index”). Even in the absence of serologic positivity, testing CSF may be helpful when central neurologic disease is suspected; a recent report noted that 15.5% of neuroborreliosis patients without evidence of serum *Borrelia* antibodies tested positive for *Borrelia* antibodies in the CSF [5]. One study found that among individuals with *Borrelia burgdorferi* antigens in the CSF, 43% had a negative intrathecal index and 20% had normal routine CSF studies; these findings indicate that antibiotics should be given even if routine CSF studies are normal when clinical suspicion for neurologic Lyme disease is high [6]. Other tests that complement the assessment but are not specific for Lyme disease include small nerve fiber skin biopsies, nerve conduction studies, neuroimaging, and cognitive testing.

Although most patients with acute Lyme disease recover completely if treated shortly after the onset of infection [7], others have residual symptoms that last months to years. Among patients who are treated with antibiotics, roughly 10–30% develop post-treatment symptoms and roughly 5–10% have residual symptoms with functional impairment (post-treatment Lyme disease syndrome or PTLDS) [8,9]. According to criteria proposed by the Infectious Diseases Society of America (IDSA) in 2006 [10], PTLDS is defined by a confirmed episode of early or late Lyme disease that meets the CDC case definition and which, despite appropriate antibiotic treatment, leads within six months to a range of symptoms that cause functional impairment. These symptoms include at least one of the following: fatigue, cognitive problems, or widespread musculoskeletal pain. The course of PTLDS may be continuous or relapsing but by this definition must span at least six months.

## 2. Neuropsychiatric Manifestations

### 2.1. Cognitive Impairment

Cognitive complaints, such as problems with word retrieval and short-term memory, occur in about 25% of patients with early disseminated Lyme disease and in up to 90% of those with PTLDS [11,12]. When tested using standardized assessments of cognitive function compared to age-adjusted norms, a smaller percent (7–30%) of people with post-treatment Lyme symptoms demonstrate objective difficulties; these deficits occur primarily in tests measuring short-term memory, verbal fluency, and processing speed [12,13,14]. It is well-established that the cognitive deficits in patients with Lyme borreliosis and with post-treatment Lyme symptoms are independent of depression severity and are on average mild in severity [15,16].

### 2.2. Psychiatric Manifestations

Severe psychiatric disturbances, such as mania or psychosis, may in rare instances be a presenting feature of acute neurologic Lyme encephalitis [17,18]. Clinical clues that suggest an underlying medical disorder such as a Lyme borreliosis include new-onset psychosis or mania in middle age, recent exposure to a Lyme endemic area or recent history of an atypical expanding rash, and accompanying clinical symptoms such as a musculoskeletal pain, atypical fatigue, joint swelling, and/or radicular, cognitive, or neuropathic symptoms. Spinal fluid studies can be informative, but it can take three to six weeks for IgG antibodies to develop and PCR assays are often negative in both early and later disease, thus accounting for misleadingly negative spinal fluid results. Another clue to initiate a search for an underlying cause is when the psychiatric condition does not respond to medications that typically would be helpful.

New-onset psychiatric symptoms in later stages of Lyme disease primarily include anxiety, mood disorders, and/or suicidality. Sensory hyperarousal to sound and/or light may also accompany psychiatric symptoms. A study using an in-depth structured psychiatric interview showed that depressive comorbidity was 4× more common in patients with definite prior Lyme disease compared to patients with medically-unexplained symptoms who self-identified as having chronic Lyme disease but lacked a proven history [19]. A retrospective chart review from a psychiatric practice of a heterogeneous group of patients with possible and confirmed tick-borne diseases revealed that 44% had a history of suicidality [20]. A case-control study comparing patients with post-treatment Lyme disease symptoms to HIV patients and healthy controls found that the rate of suicidal ideation in patients with post-treatment Lyme disease symptoms (20%) was not statistically significantly different from that in HIV patients (27%); both groups had significantly higher rates than healthy controls (5%) [21].

Many of these studies that assess the burden of psychiatric symptoms in Lyme disease were limited by small sample size, unclear diagnostic criteria, ascertainment bias (when patients are drawn from psychiatric practices or research clinics), and lack of appropriate control groups. To bypass these study limitations, two national epidemiologic studies of psychiatric illness and Lyme disease were conducted using the Danish national medical registry. A matched cohort study of all patients in Denmark between 1995–2015 (*n* = 2897) with positive *Borrelia burgdorferi* antibody production in the CSF found no increased risk of hospital-based psychiatric diagnosis or psychiatric hospitalization compared to controls. However, the study did reveal an increased risk of prescription of psychiatric medications including anxiolytics, hypnotics, sedatives, and antidepressants during the year after the positive CSF result [22]. A much larger Danish cohort study was conducted using medical records from Denmark’s entire population over a 22-year period (*n* = 6,945,837). The comparison between patients with hospital diagnoses of Lyme borreliosis and no prior psychiatric diagnosis (*n* = 12,616) and a control group of all others in Denmark showed a 28% increased incidence rate of mental disorders and a 42% increased incidence rate of affective disorders following a diagnosis of Lyme disease. The greatest increase was seen in the first year after diagnosis [23]. The analysis also revealed a 75% increase in incidence rate of completed suicide after Lyme borreliosis diagnosis and a 141% increase within three years of diagnosis. These increased incidence rates of suicide and affective disorder diagnosis persisted after removing individuals with serious comorbid medical conditions from the analyses, suggesting that psychiatric sequelae were unlikely due to co-occurring medical illness.

## 3. Neuroimaging Studies

Early structural brain imaging studies of acute neurologic Lyme disease using magnetic resonance imaging (MRI) revealed white matter changes similar to those seen in demyelinating or inflammatory disorders [24]. Controlled studies of chronic neuroborreliosis have found no differences in white matter hyperintensities in patients compared to controls [25]. More informative than structural imaging studies have been functional imaging studies of patients with post-treatment Lyme symptoms. Early studies utilizing SPECT brain imaging, which assesses blood flow, demonstrated perfusion deficits that were typically of a heterogeneous pattern. In some series, these deficits were reported to improve or resolve after appropriate treatment [26,27]. This SPECT imaging pattern is similar to what can be seen among patients with CNS lupus or cerebral vasculitis. In a study of 13 patients with Lyme encephalopathy and 9 patients with neuropsychiatric Lyme disease—patients with and without objective deficits, respectively—perfusion deficits were demonstrated in both groups but were more severe in the Lyme encephalopathy group [28]. Notably, cerebral blood flow improved after IV ceftriaxone therapy.

PET imaging has also been used to study Lyme disease by examining cerebral blood flow, metabolism, and microglial activation (a marker of inflammation). In the largest PET imaging study to date, cerebral metabolism and blood flow were compared between 35 patients with Lyme encephalopathy and 17 healthy controls [29]. Metabolism and blood flow were decreased among Lyme disease patients in gray and white matter regions, particularly in the temporal, parietal, and limbic areas. The Lyme disease group had a significantly diminished ability to enhance cerebral blood flow after a CO2 challenge (8.2 vs. 28.1%; *p* < 0.02); this reduced capacity suggests either a problem with the vascular system or a problem with neural tissue signaling for increased flow. To assess whether patients with persistent symptoms of Lyme disease have evidence of cerebral inflammation, a pilot study was conducted using TSPO-binding radioactive tracers and PET imaging among 12 patients with persistent symptoms and 19 non-Lyme controls. The study demonstrated higher binding of the radioactive tracer by microglia, the brain’s resident immune cells, in eight brain regions, supporting the hypothesis that inflammation may be involved in the pathophysiology of persistent symptoms [30].

## 4. Proposed Mechanisms of Symptom Persistence

The pathophysiology of PTLDS remains incompletely understood. However, several explanations have been proposed, ranging from persistent infection to post-infectious hypotheses.

### 4.1. Persistent Infection

Animal models demonstrate that a small number of *Borrelia* organisms can persist despite antibiotic therapy; these are usually described as viable but not culturable [31,32]. There also exist reports of persistent infection despite antibiotic therapy in humans [33,34,35], with one case describing *Borrelia burgdorferi* growing in culture [35]. The question remains as to whether persistent infection causes persistent symptoms. Studies demonstrate that the persistent *Borrelia burgdorferi* can be transcriptionally active, though cellular inflammation at the site of the persister organisms is rarely seen. A recent post-mortem case report described a 54-year-old woman with a history of doxycycline-treated erythema migrans rash and positive antibodies to *Borrelia burgdorferi* who, over 15 years, developed a neurodegenerative disease that led to her demise. Interim treatment with ceftriaxone led to partial improvement that was not sustained. Clinically and pathologically, she was diagnosed with Lewy body dementia. Post-mortem studies showed persistent *Borrelia burgdorferi* organisms in her amygdala and spinal cord [34]. While it is unclear whether these persistent organisms contributed to her neurodegenerative disease, her case does raise important questions about the pathogenicity of persistent organisms.

### 4.2. Altered Neural Activation and Dysautonomia

Like other CNS infections, *Borrelia* infection may lead to altered neural activation. This theory is supported by brain imaging studies that demonstrated altered metabolism, blood flow, and microglial activation, as described above. Specific neurotransmitter changes have not been well demonstrated. In one study, patients with CNS Lyme disease with encephalopathy showed significant elevations in the NMDA receptor agonist quinolinic acid, which may contribute to cognitive and neurologic deficits [36]. Treatment studies may also suggest a pathogenic mechanism. A small open-label study of gabapentin for patients with chronic neuropathic pain from Lyme disease led to a 90% response rate; this finding suggests that pain reduction may be coupled with the action of gabapentin, which is to bind the alpha-2-delta subunit of voltage-dependent calcium channels, which may then lead to reduced neuronal activity and neurotransmitter release [37]. A case report demonstrating marked symptom relief with ketamine, an NMDA-receptor antagonist, in a 31-year-old patient with PTLDS indicates potential alterations in glutamate pathways [38]. It would be valuable to conduct MR spectroscopy scans to assess in vivo glutamatergic signaling in a study of PTLDS, as has been done in central sensitization syndromes including fibromyalgia [39]. Exposure to *Borrelia burgdorferi* has been found to downregulate fibroblast growth factor 7 [40], a factor whose deletion is associated with reduced clustering of GABAergic synaptic vesicles; this connection raises the possibility that infection with *Borrelia burgdorferi* could affect GABA activity.

Dysautonomia is one of the major features of Long COVID [41] and may also be contributing to symptoms among some patients with post-treatment Lyme disease. In 2011, Kanjwal et al. described five patients who developed fatigue, cognitive dysfunction, orthostatic palpitations, and either syncope or near syncope that persisted after infection and treatment for Lyme disease; diagnosis with positional orthostatic tachycardia syndrome (POTS) and provision of POTS-related treatment led to significant improvement in orthostatic symptoms with four of the five patients returning to work or school [42]. In 2019, Novak et al. published a retrospective study of ten patients with PTLDS assessed for small fiber neuropathy and autonomic dysfunction. All patients had evidence of autonomic dysfunction of variable severity, usually mild-to-moderate; five patients (50%) had evidence of abnormal sweat gland nerve fiber density and nine (90%) had abnormal epidermal nerve fiber density [43].

### 4.3. Immune Dysregulation

Inflammation is a core feature of Lyme disease, as evidenced by the signs of arthritis, cranial neuritis, meningitis, radiculoneuritis, and carditis. This phenomenon is not surprising, as the outer surface proteins on *Borrelia* are 50–500× greater inducers of cytokines than lipoproteins of other organisms, such as *E. coli* [44]. Inflammation may be triggered by active infection or by remnants of infection, as indicated in the recent discovery that *B. burgdorferi* sheds immunogenic peptidoglycan during growth [45].

Ongoing immune activation has been found among patients with persistent symptoms despite antibiotic therapy, as noted above in the PET imaging study that demonstrated microglial activation [30]. Studies have shown multiple elevated immune markers in both acute Lyme disease (IL-6, IL-8, IL-12, IL-18, IFN-γ, CXCL12, CXCL13) and PTLDS (IL-6, IL-23, IFN-α, CCL19) [46,47,48,49]. A recent study indicated that in patients with acute neuroborreliosis whose symptoms persisted despite antibiotic therapy, serum IFN-α remained elevated from the initial testing at the onset of acute neurologic infection to each of the subsequent post-antibiotic treatment timepoints, with the highest levels corresponding to severe disease [50]. This finding, complemented by the observation that IFN-α levels were elevated in the CSF but then returned to normal after antibiotic therapy, supports the possibility that initial infection with *Borrelia burgdorferi* serves as a trigger for persistent symptoms due to an interferonopathy. These results align with findings from an earlier paper indicating increased genetic expression of IFN-α among patients with persistent Lyme encephalopathy [46]. One report showed that while serum CCL19 was elevated in most patients with acute Lyme disease, it was more likely to remain elevated at post-treatment follow-up visits among patients who developed PTLDS [48].

Neurologic Lyme disease, Lyme arthritis, and PTLDS have been associated with elevated levels of autoantibodies. In a study of patients with Lyme neuroborreliosis, 29% had serum IgM antibodies reactive to ganglioside and 50% had IgG antibodies reactive to cardiolipin [51]. Patients with PTLDS and Lyme arthritis have been shown to have increased anti-endothelial cell growth factor antibodies [47]. Patients with PTLDS have also been shown to have elevated anti-neuronal antibodies, with levels comparable to those seen in SLE and significantly greater than those seen in recovered Lyme disease [52]. Molecular mimicry as a mode of pathogenesis in Lyme disease is supported by several studies. Antibodies against flagellin of *Borrelia burgdorferi* have been shown to cross-react with human peripheral nerve axons [53,54,55], and antibodies against OspA peptides have been shown to cross-react with human brain, spinal cord, and dorsal root ganglia [56]. A study of individuals from Lyme-endemic areas of the northeastern USA showed elevated levels of anti-lysoganglioside GM1, anti-D1 dopamine receptor, anti-tubulin autoantibodies, and elevated neuronal cell signaling (CamKII activation) in a small cohort of patients with recurrent erythema migrans compared to controls [57]. These findings raise the possibility of an immune priming effect from repeated *Borrelia* stimulation.

## 5. Treatment Based on Presumed Mechanism of Disease

Therapeutic approaches to patients with post-treatment symptoms from Lyme disease are diverse, targeting various mechanisms of persistent symptoms.

If persistent infection is suspected, repeated antibiotic therapy should be considered. In Lyme arthritis, for example, failure of one course of antibiotic treatment is often followed by a second course of antibiotics [10,58]. In post-treatment Lyme disease, one study demonstrated significantly more responders on the primary outcome measure of fatigue in the ceftriaxone-treated group compared to the placebo group (64% vs. 18.5%, respectively; *p* = 0.001). This study also identified a potential biomarker of treatment response, as 80% of individuals with a seropositive IgG western blot treated with ceftriaxone were responders compared to only 13% of IgG western blot positive individuals treated with IV placebo (*p* < 0.01) [59]. A study of post-treatment Lyme encephalopathy found no sustained response on the primary cognitive outcome measure after 10 weeks of IV ceftriaxone, but did show sustained improvement at six months on the secondary measures of pain and physical functioning [60]; a reanalysis of this dataset indicated responder levels on the secondary measure of fatigue that were comparable to those reported in the earlier post-treatment Lyme fatigue study [61]. However, because the largest post-treatment Lyme disease study failed to demonstrate benefit from repeated antibiotic therapy [62] and because intravenous therapy carries substantial risks when administered with indwelling catheters, most international guidelines have not recommended repeated IV ceftriaxone therapy for patients with persistent symptoms.

New treatments are needed for PTLDS patients who do not respond to additional courses of antibiotic medications. Clinicians in the community are drawing upon their knowledge of other disorders or other mechanisms to help reduce symptoms among patients with PTLDS. While eradication of infection may be a goal, symptom reduction itself among those previously treated with antibiotics may lead to marked improvement in functioning and, possibly, sustained remission of symptoms. Psychiatric consultation and psychotherapy are recommended for patients with mental health sequelae from Lyme disease, as anxiety, depression, psychosis, suicidality, and/or post-traumatic stress symptoms are profoundly impairing. Mental health interventions can be an essential component of an integrated treatment plan. Suicidal thoughts or behaviors require referral to a mental health specialist to help address the hopelessness and reduce the risk of death by suicide. Immunologic or rheumatologic consultations are recommended for patients with immune dysregulation, as may be seen with persistent arthritis, new-onset autoimmune disorders, or mast-cell activation symptoms; therapies used to reduce inflammation and immune dysregulation in other related disorders such as systemic lupus or rheumatoid arthritis may be beneficial. Neurologic consultation is recommended for patients with potential autoimmune-mediated neurologic disorders such as persistent polyneuropathy or encephalitis. Physiatrists and cardiologists can be particularly helpful in the treatment of dysautonomia; medications that augment blood pressure or decrease heightened sympathetic and adrenergic responses to orthostasis may help with cardiovascular dysautonomia. Additionally, interventions that rebalance the autonomic nervous system by decreasing sympathetic activity and increasing vagal tone may be helpful; examples include breathing retraining, biofeedback to enhance heart rate variability, and possibly vagus nerve stimulation to address vagal under-activation, which is hypothesized to be a cause of dysautonomia after infection and in autoimmune disorders [63]. IV immunoglobulin (IVIg), used to treat autoimmune neuropathies including Guillain-Barre syndrome and chronic inflammatory demyelinating polyneuropathy (CIDP), may also play a role in helping patients with immune-mediated neuropathy related to Lyme disease. In a clinical series of 30 patients presumed to have *Borrelia*-triggered neuropathic pain, treatment with IVIg led to improvement in subjective clinical symptoms and objective small nerve fiber density at six months [64].

## 6. Cranial Neuropathy and Lyme Disease

Before discussing the potential role of vagus nerve stimulation in Lyme disease, it is important to highlight the impact *Borrelia burgdorferi* has on cranial nerves. Cranial nerve involvement occurs in roughly 50–80% of all patients with neuroborreliosis [65]. Facial nerve palsy is by far the most common, accounting for roughly three quarters of Lyme-associated cranial neuropathies [66]; this manifestation is most often unilateral but can also be bilateral [67]. Although involvement of the seventh cranial nerve is widely recognized as a feature of neurologic Lyme disease, case reports exist documenting that *Borrelia burgdorferi* infection can affect nearly every cranial nerve. While deficits often appear in isolation, they may also appear in coordination with other cranial neuropathies as a mononeuropathy multiplex [68]. Optic neuritis is rare and usually presents with painless, moderate, progressive vision loss [69], though sudden onset of bilateral vision loss has been documented [70]. Isolated oculomotor palsy has been described in case reports of both pediatric and adult patients [71,72,73]; in all cases, deficits resolved with antibiotic treatment. Trochlear nerve palsy has been documented in isolation as well as in coordination with facial nerve palsy and with abducens nerve palsy [74,75,76,77]. One report documented unilateral MRI-confirmed trigeminal nerve inflammation with mild ipsilateral maxillary hypoesthesia, which resolved after antibiotic treatment [78]. Individual case reports exist of Lyme disease presenting with Raeder syndrome, a complex of Horner syndrome accompanied by ipsilateral deficits in the trigeminal nerve distribution [79,80]. Abducens nerve palsy has been documented in isolation as well as with trochlear involvement, with optic nerve perineuritis, and with oculomotor palsy [81,82,83,84]. Vestibulocochlear nerve involvement is rare and can lead to hearing loss and vertigo [85], with longer infection leading to greater likelihood of permanent hearing deficits [86]. Involvement of the recurrent laryngeal nerve, a branch of the vagus nerve, is extremely rare; deficits are typically unilateral and cause hoarseness or sore throat, though in rare instances bilateral involvement can necessitate supplemental oxygen [87] or mechanical ventilation [88]. Lyme disease may also directly affect the vagus nerve, as suggested by impaired respiratory modulation of cardiac vagal tone in a cohort of Lyme patients compared to controls [89]. There exists one case report of a 19-year-old individual with unilateral accessory nerve involvement in coordination with involvement of the brachial plexus and recurrent laryngeal nerve [90], as well as one case report of probable hypoglossal and vagal paralysis in an infant with lingual asymmetry, difficulty swallowing, and dysphonia [91].

## 7. Vagus Nerve Stimulation

Vagus nerve stimulation (VNS) may be a treatment with considerable promise for patients with post-treatment Lyme disease, as research over the past two decades has demonstrated that VNS can have multiple salutary effects in both animal models of disease and human illness. VNS is a technique within the umbrella category of neuromodulation, which also includes transcranial magnetic stimulation (TMS), transcranial direct current stimulation (tDCS), focused ultrasound, and deep brain stimulation (DBS). To our knowledge, none of these techniques have yet been studied among patients with persistent Lyme disease symptoms. The vagus nerve plays a critical role in parasympathetic control of the heart, lungs, and digestive tract, and VNS has been shown to improve mood, decrease pain, and decrease inflammation in various human studies [68,69].

Given that Lyme disease is characterized by inflammation as well as multi-system and neuropsychiatric symptoms and given that patients who have had adverse reactions to medications may prefer non-pharmacologic approaches, it is valuable to consider the potential role of VNS in the amelioration of post-treatment Lyme disease symptoms. Given the importance of glutamate [92] and GABA [93] neurotransmission in the dorsal motor nucleus of the vagus nerve, and given the possible effect of *Borrelia burgdorferi* infection on glutamate [38] and GABA [40] activity, there is a potential mechanism by which modulation of the vagus nerve could address symptoms from *Borrelia* infection.

The vagus nerve, which contains both afferent and efferent fibers, is the 10th and longest cranial nerve. Surgically-implanted VNS was approved by the FDA to treat refractory epilepsy in 1997 and to treat refractory depression in 2005 [94,95]. Because of the cost and risks associated with surgically implanted VNS, it has not been widely used as a clinical intervention. However, this paradigm has changed considerably since the development of external transcutaneous vagus nerve stimulators.

### 7.1. Transcutaneous VNS

Two external sites are the main foci of current transcutaneous VNS studies—the cervical and auricular locations. Stimulation of the cervical cardiac branch of the vagus nerve using a hand-held device received FDA approval for the treatment of migraines in 2018 and for cluster headaches in 2019. While the ear has been the focus of auricular acupuncture for over 2500 years, transcutaneous auricular VNS (taVNS) was only first described in 2000 [96]. The external ear hosts the auricular branch of the vagus nerve, innervating the tragus and the cymba concha as well as the crura of the antihelix and the crus of the helix; these superficial locations enable external stimulation without surgery. Several fMRI studies have reported that taVNS is associated with activation of central nervous system structures that have anatomical projections from the vagus nerve: the nucleus of the solitary tract, locus coeruleus, nucleus accumbens, dorsal raphe, thalamus, and others [97,98,99,100,101]. These findings confirm that electrical stimulation of the auricular branch of the vagus nerve sends signals directly to the vagal nuclei in the medulla, thereby providing a direct pathway from the skin to the central and autonomic nervous systems and a potential mechanism for the diffuse central and peripheral effects of taVNS.

Over 400 clinical studies of vagus nerve stimulation are currently listed on clinicaltrials.gov, with 49% (*n* = 197) being studies of non-invasive transcutaneous stimulation. The topics under investigation include epilepsy, depression, obesity, headaches, inflammatory bowel disease, rheumatoid arthritis, post-stroke recovery, heart failure, and atrial fibrillation. “Long-hauler” syndromes triggered by infection, such as “Post-Acute Sequelae of COVID-19” (PASC), are also being studied.

Over the last decade, multiple studies have assessed the safety, feasibility, and efficacy of this method in different populations, generally indicating relative safety and varying effect sizes for different diseases [102,103]. Unlike surgically-implanted VNS, taVNS is emerging as an easy-to-administer and relatively safe way to modulate the vagus nerve system, with recent studies indicating that it can be self-administered safely at home [104].

### 7.2. Preclinical Studies

Preclinical trials show that vagus nerve stimulation generates an anti-inflammatory effect mediated by the splenic nerve that has been named the “anti-inflammatory reflex” [105,106]. In animal models of endotoxic shock, bilateral cervical vagotomy resulted in increased acute inflammation with decreased corticosteroid response and an increased TNF response. Hepatic and serum TNF response, however, significantly decreased in vagotomized animals with electrical stimulation of the distal remains of the severed VN compared with sham-operated animals [107]. Significant reductions in plasma inflammatory markers including TNF-α and IL-1β have been shown in mouse studies of minimally invasive VNS [108] and transcutaneous VNS [109].

Vagus nerve stimulation has not only peripheral but also central anti-inflammatory effects. It was recently shown in a murine model that specific neurons in the afferent and motor nuclei of the vagus nerve are activated in response to lipopolysaccharide (LPS) and can exert control over behavioral responses known as “sickness behavior” [110]. VNS also was shown to prevent LPS-induced hippocampal microglial activation [108].

Stimulation of the sciatic-vagal network with electroacupuncture was studied in a mouse model of Lyme arthritis. Mice infected with *B. burgdorferi* were stimulated for two weeks with a 10-min stimulation consisting of an alternating current of 40 mA, a frequency of 10 Hz, and a pulse width of 50 s. At the week four evaluation, leukocyte infiltration in the joints and systemic inflammatory cytokines were reduced in all stimulated mice in comparison with sham-treated mice, suggesting that electroacupuncture may be of adjunctive benefit to reduce inflammation in various manifestations of Lyme disease including PTLDS [111].

### 7.3. Clinical Use

Implanted VNS has been shown to be safe in many diseases, including human inflammatory diseases such as rheumatoid arthritis (RA) and systemic lupus erythematosus (SLE). An open-label study of RA demonstrated that surgically-implanted VNS has both biomarker and clinical effects, inhibiting the production of inflammatory cytokines (TNF, IL-1β, and IL-6) and attenuating arthritis severity [112]. A subsequent open-label pilot study of transcutaneous cervical VNS for RA showed that patients with high disease severity showed improvement in disease severity as well as reductions in C-reactive protein and IFN-γ after only four days of treatment [113]. A randomized, double-blind, sham-controlled trial of short-duration taVNS among 18 patients with SLE found that in addition to being safe and well-tolerated, taVNS led to a greater reduction in joint pain and fatigue as well as reduced joint tenderness on clinical examination compared to masked taVNS [114]. As indicated in preclinical studies, clinical pilot studies have demonstrated antinociceptive effects of both invasive and non-invasive VNS [115].

Stimulation effects are not limited to modulation of inflammatory processes. Vagus nerve stimulation in epilepsy patients showed associations with mood improvements [116], helping lead to FDA approval for depression in 2005 [117]. A meta-analysis of six clinical trials with more than 1500 individuals showed that VNS combined with treatment as usual resulted in greater remission rates from depression (based on MADRS scores), with effects more likely to be sustained in the long term, compared to treatment as usual alone [118]. The mechanism of the antidepressant effect of VNS is uncertain, but it is hypothesized that VNS enhances monaminergic neurotransmission and neural plasticity [119]. Multiple studies have shown that VNS improves noradrenaline and serotonergic neurotransmission in brain regions known to be impacted by depression [120].

Trials utilizing at-home administration of taVNS demonstrate not only the convenience of this technology but also its potential to reduce symptoms like fatigue and pain in patients with multi-system illness syndromes. A recent pilot study among individuals with Long COVID demonstrated that taVNS can be delivered safely at home with completely remote instruction and monitoring [104]. In a study of fatigue in primary Sjogren’s syndrome, patients were able to conduct a long-term (52 days) treatment at home using cutaneous VNS twice daily [121]. Similarly, a 10-week course of transcutaneous VNS administered at home was shown to be feasible for veterans suffering from pain and migraines [122]. In each study, patients reported symptomatic relief (in fatigue and pain respectively) at the end of treatment. Preliminary data from an open-label pilot study of at-home taVNS treatment for Long COVID with chronic fatigue syndrome showed that 8 of the 14 patients fulfilled an a priori definition of symptomatic improvement after six weeks of treatment [123].

A systematic review of quality and evidence in taVNS clinical trials suggested that implanted and transcutaneous VNS demonstrate similar clinical and biological impact in reducing inflammatory markers like TNF and IL-1β [102]; while each study individually has methodologic limitations, the results support this conclusion when taken together. This evidence makes taVNS an excellent candidate for testing in groups of patients with multi-system illness manifestations and “long-hauler” syndromes such as Long COVID, PTLDS, and Myalgic Encephalomyelitis/Chronic Fatigue Syndrome.

### 7.4. Safety

A review of 32 studies of transcutaneous auricular VNS (among 51 studies of transcutaneous VNS) concluded that taVNS is a safe and well-tolerated treatment, with only 15.6% of the 701 patients experiencing one or more side effects [103]. The most common side effect from taVNS is local skin irritation. Although these safety results are encouraging, it is important to realize that safety results from clinical trials may not be generalizable to the general population. For safety reasons, most studies exclude people from transcutaneous VNS studies who have cardiovascular disorders, metal implants above the neck, or a recent history of symptomatic bradycardia or vasovagal syncope. The safety profile of taVNS for the larger non-research community has yet to be described.

### 7.5. Limitations of Transcutaneous VNS

Vagus nerve stimulation has proven to be an effective treatment in its implanted form. The less invasive form of transcutaneous stimulation (taVNS) is relatively new and has yet to be proven consistently effective [102]. Despite targeting the same nerve, non-invasive and invasive forms of VNS differ in several respects:

(a) The branches of the VN differ widely in size. The myelinated Aβ axons of the cervical branch, which is the site of surgically implanted stimulation, are five to six times thicker than those in the auricular branch of VN. The latter axons are much smaller (with less than 30% having a diameter > 7 nm) and sometimes cannot be appropriately anatomically defined [124,125].

(b) The duration of the stimulation depends on the method of administration. Invasive VNS is a long-term treatment and usually lasts for months to years. Non-invasive VNS is much safer and less costly for a patient, but the average duration of each stimulation session ranges from just minutes to two hours with clinical trial duration for each participant lasting from days to months [103].

(c) Individual variability exists in response to stimulation parameters. Intracranial recordings done in seven surgery patients with implanted and non-invasive vagus nerve stimulations showed that invasive VNS is modulated by frequency whereas transcutaneous auricular VNS is more affected by the amplitude of stimulation. In both invasive and non-invasive versions, effects of stimulation may vary across individuals and modalities [126].

(d) Despite an increasing number of studies of transcutaneous VNS, there is no consensus on many parameters of the stimulation, including the optimal location [127]. In any given study a researcher may change frequency, waveform, amplitude, electrode size, and other parameters that all may affect the outcome [128]. To our knowledge, there have been no systematic studies that have evaluated which parameters or combination of parameters have the greatest influence on taVNS efficacy.

## 8. Conclusions

Post-treatment Lyme disease can be a highly debilitating condition with prominent fatigue, pain, and cognitive problems as well as other infection-associated chronic symptoms. Given that Lyme disease is increasing in prevalence and that 10–30% of patients experience persistent symptoms, the number of persistently symptomatic patients will likely continue to increase internationally. The multi-system toll on the individual can be life-altering. New treatments are needed for those patients with persistent symptoms who no longer benefit from antibiotic therapy. The above review of transcutaneous VNS reporting safety and beneficial effects for pain, depression, fatigue, and inflammation is encouraging. However, there is more work to be done regarding identification of the optimal location for stimulation and the optimal parameters for stimulation intensity, duration, and frequency. If well-controlled double-blinded studies demonstrate that VNS is helpful for patients with “long-hauler” syndromes such as post-treatment Lyme disease, VNS will be a welcome addition to the treatment options for these conditions.

## Data Availability

Not applicable.
